# *Yucca schidigera* Usage for Healthy Aquatic Animals: Potential Roles for Sustainability

**DOI:** 10.3390/ani11010093

**Published:** 2021-01-06

**Authors:** Bilal Ahamad Paray, Mohamed F. El-Basuini, Mahmoud Alagawany, Mohammed Fahad Albeshr, Mohammad Abul Farah, Mahmoud A. O. Dawood

**Affiliations:** 1 Department of Zoology, College of Science, King Saud University, P.O. Box 2455, Riyadh 11451, Saudi Arabia; bparay@ksu.edu.sa (B.A.P.); or albeshr@ksu.edu.sa (M.F.A.); mfarah@ksu.edu.sa (M.A.F.); 2 Faculty of Desert Agriculture, King Salman International University, South Sinai 46612, Egypt; m_fouad_islam@yahoo.com; 3 Department of Animal Production, Faculty of Agriculture, Tanta University, Tanta 31527, Egypt; 4 Poultry Department, Faculty of Agriculture, Zagazig University, Zagazig 44511, Egypt; dr.mahmoud.alagwany@gmail.com; 5 Department of Animal Production, Faculty of Agriculture, Kafrelsheikh University, Kafrelsheikh 33516, Egypt; 6 Center for Applied Research on the Environment and Sustainability, the American University in Cairo, New Cairo 11835, Egypt

**Keywords:** aquaculture development, phytogenic, *Yucca schidigera*, feasibility, added value

## Abstract

**Simple Summary:**

This review presents an updated and exclusive collection of results about yucca’s beneficial effects as phytogenic additives for clean aquaculture activity. The overall performances of aquatic organisms treated with yucca as dietary additives of water cleaners encourage performing further studies to prove its mode of action based on biochemical and biological techniques.

**Abstract:**

In modern aquaculture systems, farmers are increasing the stocking capacity of aquatic organisms to develop the yield and maximize water resources utilization. However, the accumulation of ammonia in fishponds regularly occurs in intensive aquaculture systems, resulting in reduced growth rates and poor health conditions. The inclusion of yucca extract is recognized as a practical solution for adsorbing the waterborne ammonia. Yucca has abundant amounts of polyphenolics, steroidal saponins, and resveratrol and can be used as a solution or as a powder. In this context, this review aimed to investigate the possible regulatory roles of yucca extract on aquatic animals’ performances. Concurrently, the feed utilization, growth performance, and physiological status of aquatic species can be improved. Additionally, the yucca application resulted in enhancing the antioxidative, immunological, and anti-inflammatory responses in several aquatic animals. Exclusively, the present review proposed a protective solution through the application of yucca extract in the aquafeed and rearing water of aquatic animals suffering from ammonia accumulation. Furthermore, it shows how yucca could enhance the growth, survival rates, blood biochemical quality, immunological indices, and the antioxidative capacity of aquatic animals in light of the relevant published data.

## 1. Introduction

With the recent global environmental changes (e.g., global warming and pandemics), the necessity to produce organic and healthy food for humanity [1], and the need to reach food sustainability objectives that focus on finding alternative food sources with feasible and applicable practices [2], tremendous efforts are requested from primary animal protein producers (poultry, livestock, and aquaculture sectors) to guarantee sufficient protein amounts at low cost [3]. Recently, food security agencies have called for limiting the usage of antibiotics and chemotherapies in poultry, livestock, and aquaculture production due to their negative impact on natural immunity, either in the animals or the human body and their hazardous environmental risks [4,5,6]. Therefore, using natural alternative substances that act as growth promotors, immunostimulants, and antioxidative agents is urgently needed [7]. Medicinal herbs and their extracts are successfully applied as feasible and environmentally friendly products in the animal nutrition field [8].

*Yucca schidigera* and its extracts are among the medicinal plants associated with plenty of beneficial effects when applied in aquaculture [9]. Several studies found that aquatic animals’ performances showed improvements as a direct result of using yucca as feed or water additives [10,11,12,13,14]. Dietary yucca increases protein metabolism in the fish body, with a possible reduction in ammonia excretion [9]. The improvement in protein metabolism enhances feed utilization and results in a high feed intake and growth rate [15]. Therefore, using dietary yucca results in improving the health condition, immune, and antioxidative responses of aquatic organisms [16]. Yucca plant originates from the deserts of the Southwestern United States and Mexico, which is known for their high temperature, lack of water, and stressful conditions [10]. Concurrently, yucca is characterized by its anti-stressor potential and beneficial effects that make it a prospective phytogenic additive for the aquaculture industry [12,17]. Yucca has abundant amounts of saponin and resveratrol, which can eliminate the waterborne ammonia and lower its impacts on aquatic animals’ performance and health [18]. Therefore, many commercial aquatic products include yucca and saponin in their formulation to be applied in aquaculture ponds and intensive systems. It enhances the water quality, feed intake, growth rate, anti-oxidative, and immune responses in aquatic species. Furthermore, yucca increases resistance against infectious bacteria and invaders [13].

Recently, aquaculture activity has become massively developed and a safe and unique choice to produce cheap and nutritious seafood products as animal protein sources [19,20]. In this regard, intensive systems are applied to produce the maximum possible amounts of aquatic biomass per unit of water resources [21]. Nevertheless, the intensification of production induces side effects on aquatic animals’ performances due to the high stocking capacity, ammonia emissions, leftover feed, lack of dissolved oxygen, feces, and organic materials [20,22]. These stressful conditions reduce feed consumption, growth rate, immunity, and antioxidative responses, with possible opportunities for bacterial infection [23,24]. Several managerial efforts to enhance water quality are normally applied, including regular water exchange, injection with oxygen, using pedals, filtering water with mechanical and biological filters, and including some nitrifying microorganisms [25]. Additionally, functional herbal substances, such as feed or water additives, are strongly recommended as environmentally friendly additives [26,27].

The present review aimed at surveying the available outputs of studies that investigated the application of yucca in aquatic organisms as an alternative option for aquaculture sustainability. These studies’ results are discussed and recommendations are made to provide clear guidelines for yucca application, either as a feed additive or water supplement.

## 2. The Nature, Sources, and Composition of Yucca

The *Yucca* genus comprises perennial nature woody flowering shrubs and trees with various sword-shaped leaves [28], belonging to the family Asparagaceae and subfamily Agavoideae [29], which encompasses about 49 species spread particularly in arid regions of the American South West and Mexico and mostly producing economic benefits, including ornamental, industrial, nutritional, and medical applications [10,30,31].

Yucca products (powder and juice) are commercially available, where they were approved in 1965 by the Food and Drug Administration (FDA) (21 CFR 172.510) and can be used as dietary additives or supplements due to their beneficial impacts on well-being, growth performance, nutrient utilization, the removal of fecal odors and ammonia, hydrogen sulfide, and some other hazardous volatile compounds in human and animal excreta [32,33]. The main constituents of yucca powder or extract (YE) are steroidal saponins, polysaccharides, and polyphenols, which possess antioxidant, anti-inflammatory, antiviral, antiprotozoal, antiplatelet, antimutagenic, anticancer, cholesterol reduction, and iNOS-expression-inhibiting activities [34].

Saponin (triterpenoid saponins) originated from *Quillaja saponaria* (Molina: widely found in Peru, Chile, and Bolivia) and differs from saponin with a yucca origin (steroidal saponins), and is mainly used as an adjuvant in veterinary vaccines [18]. Saponins are one of the phytochemical compounds in the yucca and Quillaja plants that consist of two parts: one is hydrophobic (a lipophilic nucleus, namely, the sapogenin) and the other is a hydrophilic carbohydrate side chain(s) or oligosaccharides, which explains the surfactant ability of these compounds [35,36]. Saponin binds with cholesterol in an insoluble compound that results from a lipophilic bond between the hydrophobic parts of saponins (sapogenin) and cholesterol (hydrophobic steroid or triterpenoid nucleus) in a stacked micellar aggregation, thus inhibiting the recycling of entero-hepatic cholesterol [36,37]. Saponins have a direct impact on the permeability of intestinal cells, as well as the gastro microbiota (antiprotozoal activity) by forming complexes with sterols (cholesterol) in cell membranes [38,39]. Yucca phenolic constituents include two stilbenes with antioxidant and anti-inflammatory potentials, where the first is yuccaol A, B, C, D, and E (trans-3,3’,5,5’-tetrahydroxy-4’-methoxystilbene) and the second is resveratrol (trans-3,4’,5 tetraxydroxystilbene) [34,40,41].

## 3. Yucca as a Growth Promotor

The application of plant-based products to support the growth of aquatic organisms has become widely used [15,26] (Table 1). Dietary incorporation of yucca products has favorable effects on the growth performance, feed efficiency, and health of aquatic animals [42,43]. The improvement in growth as a result of yucca supplements may be linked to the enhancement in water quality and feed utilization, which relies on the intestinal status via modulating the gut flora, enzyme activity, and absorption [33,44,45].

In this context, Wang et al. [42] evaluated the effect of dietary incorporation of *Y. schidigera* extract (YSE) at different levels of 0, 200, or 400 mg/kg diet for 8 weeks on the growth performance of mirror carp (*Cyprinus carpio*) with an initial weight of 45.21 ± 0.43 g. The growth results of this study showed an enhancement in the final body weight and weight gain rate with YSE at 400 mg/kg diet compared to the control group. The boosted growth performance was linked to the alteration in the microbial population, which enhanced the feed digestion and utilization, regardless of the non-significant alteration in intestinal digestive enzymes. Moreover, Peterman et al. [46] found a remarkable growth performance (higher weight gain and specific growth rate (SGR)) and feed utilization (lower feed conversion ratio (FCR)) after a 3 month feeding period in channel catfish (*Ictalurus punctatus*, 11 g initial body weight) fed a commercial blend (One Current™) of prebiotic fiber, oregano, thyme, cinnamon, essential oils, and *Y. schidigera* compared to the control group.

Furthermore, El-Keredy and Naena [47] studied the growth of Nile tilapia (*Oreochromis niloticus*, initial weight of 20 g) infected with *Pseudomonas aeruginosa* in response to the dietary supplementation of YSE at levels of 0.1, 0.14, and 0.2% for 8 weeks. The fish group fed 0.1% YSE showed the higher growth performance, feed intake (FI), feed efficiency ratio (FER), and protein efficiency ratio (PER); meanwhile, the groups that received high levels of YSE (0.14 and 0.2%) showed no significant alteration compared to the control. The authors demonstrated that high levels of YSE in diets increases the saponin concentration, which negatively affects nutrient absorption; this is in line with the results of Chen et al. [48] for juvenile Japanese flounder (*Paralichthys olivaceus*) a fed lower saponin content (originated from soybean) in their diet, which resulted in increased growth due to the high absorption rate from the intestine that was motivated by the slight permeabilization due to saponins. This would elucidate why a low concentration (<1000 ppm) of saponin-rich plant extracts can exert a favorable influence on fish growth, whilst high levels participate in the development of enteritis. In this context, Bae et al. [49] reported no significant differences in the growth and feed efficiency between olive flounder (*P. olivaceus*, 5.26 ± 0.17 g) that were fed yucca meal for 8 weeks at the level of 1.5 g/kg diet and the control group. Likewise, Tidwell et al. [50] reported a reduction in body weight and elevation in the FCR of juvenile channel catfish (*I. punctatus*) fed YSE at the level of 1.1 g/kg diet.

In a different approach, Abdel-Tawwab et al. [12] examined Nile tilapia’s (28–32 g) responses to YSE and/or the yeast *Saccharomyces cerevisiae* as water additives at the level of 1 g/m^3^ for 8 weeks. The results of the Nile tilapia growth were improved (*p* < 0.05) due to the water additives and the highest growth was recorded in fish treated with YSE + yeast. Likewise, Fayed et al. [16] studied the effect of three concentrations of YSE (0.25, 0.50, and 0.75 mg/L) in the water on European seabass juveniles (*Dicentrarchus labrax*, 5.0 ± 0.5 g) for 45 days. The growth performance increased (*p* < 0.05) with increasing YSE levels in the water in association with the hematologic and water quality improvement.

Furthermore, Elkhayat et al. [45] studied the responses of European seabass (*D. labrax*) with an initial weight of 5.00 ± 0.5 g to YSE supplementation at concentrations of 0, 0.25, 0.50, or 1.00 g/kg diet. The results of the 45-day feeding trial showed amelioration in weight and FCR, particularly at the level of the 1.00 g/kg diet. Likewise, Njagi et al. [13] examined the effect of dietary Yucca meal (YMS) at levels of 0, 0.1, 0.3, 0.5, 1.0, and 2.0% on the growth of Nile tilapia (*O. niloticus*, 3.8 ± 0.05 g) for 10 weeks. The results of this trial showed an improved growth performance and whole-body protein content with 0.1% YMS compared with the control. In addition, Angeles Jr. et al. [51] investigated the impact of a 6-week feeding trial with *Q. saponaria* and/or extracts at the level of 150 mg/kg diet on Nile tilapia’s (*O. niloticus*, 1.9 ± 0.08 g initial body weight) growth and survival rate. The growth results of the fish group fed an extracts mixture of 150 mg/kg *Q. saponaria* + 150 mg/kg *Y. schidigera* were significantly higher compared to the control groups. However, no differences (*p* > 0.05) in survival rates was found between the groups. Consistent with these results, Güroy et al. [52] performed a 12-week feeding trial to determine the impact of YSE (0, 0.075, 0.1, or 0.15%) in a practical diet on the growth of striped catfish (*Pangasianodon hypophthalmus*, initial mean weight 1.78 ± 0.05 g). The dietary incorporation of YSE at a high level of 0.15% improved the SGR and decreased the FCR.

Moreover, Gaber [33] examined the full substitution of fish meal protein in the control group (FMC) with the meal of soybean, cottonseed, sunflower, or linseed supplemented with YSE on the growth of Nile tilapia (*O. niloticus*) with an initial weight of 14.2 ± 2.9 g for 6 months. The results of the YSE supplementation with plant-protein-based diets exhibited growth performances that did not differ (*p* > 0.05) from that of fish fed FMC + YSE while differing (*p* < 0.05) from the control and linseed meal (LSM) diets. All groups fed diets with YSE showed a higher apparent protein digestibility coefficient, whole-body protein content, and lower whole-body lipid content compared to the control. Moreover, El-Saidy and Gaber [53] examined the dietary supplementation of graded levels of *Y. schidigera* powder (0, 250, 500, 750, 1000, 1250, or 1500 mg/kg diet) on *O. niloticus* that were about 16.82 g (initial body weight). The results of the 30-week feeding trial showed that *Y. schidigera* supplementation at levels of 750 or 1000 mg/kg diet enhanced the final body weight (FBW), weight gain (WG), SGR, FER, and PER compared to the control group. Fish groups that received diets with yucca exhibited a higher apparent digestibility coefficient (ADC) of protein and lipid.

In addition, Kelly and Kohler [43] investigated the impact of a feeding regime with YSE (0, 0.01, or 0.1 g/kg diet) on the growth performance of post-yolk-sac and juvenile channel catfish (*I. punctatus*). After the 12-week feeding period, post-yolk-sac channel catfish fry fed the YSE had the highest weight gain compared to the control. Furthermore, Francis et al. [44] tested the dietary supplementation (0, 150, or 300 mg/kg diet) of a saponin mixture on common carp (*C. carpio* L.) with a 19 g initial weight for 8 weeks. The highest weight gain and protein utilization were observed with 150 mg/kg compared with other groups. The best results for the FCR and metabolic growth rate were with the 150 mg/kg supplementation. In addition, Francis et al. [54] studied the incorporation of a Quillaja saponin (QS) mixture at the level of 0, 150, or 300 mg/kg in the diets of tilapia (*O. niloticus*) for 14 weeks. The results of the tilapia performance that showed the highest weight gain, average values for energy retention, and apparent lipid conversion were in the group supplemented with 300 mg/kg.

For shrimp cultivation, Hernández-Acosta et al. [55] examined Pacific white shrimp (*Litopenaeus vannamei*, 2.6 g initial body weight) cultured in low-salinity water and fed diets with *Y. schidigera* and *Q. saponaria* extracts (NTF) at the levels of 0, 0.25, 0.50, 1.00, and 2.00 g/kg diet for 40 days; they found significant results regarding the weights and FCR with 1.00 and 2.00 NTF compared to other groups. The increase in weights and decrease in FCR in response to *Y. schidigera* and *Q. saponaria* supplementation may be due to increased protein synthesis, digestive enzymes, and promotion of nutrient absorption. Moreover, Yang et al. [11] investigated *L. vannamei* (0.82 ± 0.02 g initial body weight) that were fed diets with YSE at the levels of 0, 0.1, 0.2, or 0.3% for 100 days and reported that the addition of 0.2% YSE in the diet was beneficial to the growth and the quality of water and attributed this enhancement in the growth to YSE steroidal saponins and other surface-active substances, which promoted nutrient absorption.

## 4. Yucca as an Immunostimulant

One of the determinants of aquaculture is the outbreak of diseases, and chemotherapy that includes antibiotics is one of the main methods of disease control; however, due to the heightened apprehension about the effects of long-term use (e.g., the production of resistant pathogens strains, aggregation of toxic residues, depletion of the immune system, and environmental hazards), their use in different countries has been discouraged [56,57]. Currently, eco-friendly natural strategies and/or alternatives to antibiotics, such as medicinal herbs and beneficial microorganisms (probiotics, prebiotics, and synbiotics), have become an area of interest of many studies [58,59,60].

The efficacy of yucca as a natural immunostimulant substance is attributed to its high content of bioactive components (e.g., alkaloids, terpenoids, saponins, steroids, phenolics, tannins, glycosides, and flavonoids) [36]. Importantly, treatment with yucca results in an enhanced immune response along with an activated antioxidative capacity (Table 2). The aquatic organisms treated with dietary yucca showed direct enhanced local intestinal immunity, which was correlated with a general immune system enhancement due to the influence of yucca as an antibacterial agent against pathogenic microorganisms in the gastrointestinal tract (GIT) [42,61]. Accordingly, yucca reduces the GIT inflammation that is induced by pathogens and toxins and relieves the stress that results from unfavorable aquaculture conditions [13]. Subsequently, treatment with yucca enhances aquatic animals’ resistance against infection by pathogenic microorganisms that may attack the organism in the ponds [13]. Indirectly, when the rearing water is treated with yucca extracts, the accumulation of ammonia is diminished and reduces the stressful impacts on fish [12], which could lead to immunosuppression and pathogenic invaders attacking if continued for a long time.

In this context, Wang et al. [42] indicated that YSE dietary incorporation could boost the immunity of mirror carp (*C. carpio*) by increasing the lysozyme activity (LYZ) with the inclusion of YSE at 200 and 400 mg/kg diet and increasing C3 and C4 by using YSE at 400 mg/kg diet. Furthermore, YSE supplementation up-regulates the intestine TGF-β2 mRNA levels and downregulates the mRNA levels of TNF-α, IL-1β, and IL-6. The authors referred to the YSE saponin content and its ability to form immunostimulatory complexes to explain the enhancement in immunity. In the same manner, Bae et al. [49] found that yucca meal at 1.5 mg/kg diet as a functional additive in olive flounder (*P. olivaceus*) diets improved nitroblue tetrazolium (NBT) results and the cumulative survival rate compared to the control group. Likewise, El-Keredy and Naena [47] reported lower mortality and morbidity rates for Nile tilapia (*O. niloticus*) infected with *P. aeruginosa* in all YSE groups compared to the control, where the best survival percentage was recorded in the group that was fed 0.1% YSE. In addition, the enhancement in immunity was linked to the impacts of the active components in YSE. Similarly, Peterman et al. [46] demonstrated that fish fed One Current™ displayed a higher survival rate compared to the control group.

Regarding Nile tilapia, Abdel-Tawwab et al. [12] reported an improvement in blood profile (increasing serum total lipids, total protein, albumin, and globulin, and decreasing aspartate aminotransferase, uric acid, and creatinine) in response to YSE and/or the yeast *S. cerevisiae* as a water treatment. Moreover, Njagi et al. [13] found an improvement in the immunity of Nile tilapia (*O. niloticus*) in terms of plasma LYZ activity, respiratory burst activity (NBT), and resistance against *Aeromonas hydrophila* with the addition of YMS at the level of 0.1% and linked the obtained results to the ability of saponin to form immunostimulatory complexes. Furthermore, Amoah et al. [62] evaluated the impact of the dietary additives of 0.4% Song-gang^®^ stone (SG), 0.05% Yucca meal (YM), and 0.05% β-glucan (BG) on the innate immunity of juvenile Amur catfish (*Silurus asotus*) with an initial weight of 4.95 ± 0.05 g for 8 weeks. The results of the lysozyme activity were higher in fish fed the SG, YM, and SG + BG diets than those of fish fed the control diet. All experimental groups showed no adverse impacts in terms of blood health. Similarly, Güroy et al. [52] concluded that the dietary incorporation of YSE at a high level of 0.15% improved some hematological parameters of striped catfish (*P. hypophthalmus*). Moreover, Fayed et al. [16] found that the presence of YSE in water improves the blood profile and immunity of European seabass juveniles in terms of white blood cells count (WBCs), red blood cells count (RBCs), hematocrit (Hct), hemoglobin (Hb), lymphocytes, and neutrophils, as well as plasma LYZ activity, particularly at a high level of yucca extract (0.75 mg/L). Similar results were obtained by Reham et al. [63] for Nile tilapia (*O. niloticus*, 51 g initial weight) hematology following a YSE implementation in water for 93 days.

For shrimp, Yang et al. [11] found that the addition of 0.2% YSE in the diet was beneficial to shrimp immunity and linked this improvement to increased serum total protein and reduced harmful and volatile chemicals. Likewise, Yeh et al. [64] found that exposing giant freshwater prawns (*Macrobrachium rosenbergii*) (17.9 ± 2.7 g) to saponin in the water at different concentrations of 0, 0.3, 0.6, 0.9, and 1.2 mg/L for 168 hrs resulted in increasing the immunity in terms of the total hemocyte count (THC) and decreased the phenoloxidase activity and the respiratory protein level, particularly at 0.9 mg/L.

From a different point of view, saponins that originated from Quillaja and Yucca at high concentrations in water or diets can be considered lethal. The addition of Quillaja saponins to water at a concentration of 4000 ppm did not lead to the deaths of carp within 18 h; meanwhile, the addition of Yucca saponins at a concentration of 1000 ppm led to the death of all fish within 18 h. Moreover, Quillaja saponin at 2000 mg/kg in standard diets of the first-feeding stage induced high mortality of tilapia larvae [8]. Similarly, Quillaja and Yucca saponins at very low concentrations in water (20 ppm) killed 40 and 20% of *Biamphalaria glabrata* snails, respectively [65].

Moreover, a saponin from soybean shows negative impacts on aquatic animals’ growth [66], as previously reported in Atlantic salmon (*Salmo salar*) [67,68], Japanese flounder (*P. olivaceus*) [48], gilthead sea bream (*Sparus aurata*) [69], and European sea bass (*D. labrax*) [70].

## 5. Yucca as an Antioxidative Agent

Oxidative stress is one of the major concerns in aquaculture [71,72], which occurs due to the imbalance in reactive oxygen species (ROS) production and the disposal process [73,74]. The liver, intestine, and kidneys are the main components of the antioxidant system due to their function in the disposal of ROS by producing a number of key enzymes, such as superoxide dismutase (SOD), catalase (CAT), and glutathione peroxidase (GPX) [75]. The antioxidant defense system is tightly linked to the health status and the immune system of fish [76]. The aquatic animal antioxidant system is vulnerable to biotic and abiotic factors (e.g., phylogenetic position, age, physiological condition, presence of xenobiotics, environmental factors, diets, and feeding behavior) [77]. Medical plants, with their numerous active components, are thought to possess different functions as immunostimulants and antioxidants [78,79,80,81]. The Yucca plant and its products have shown antioxidant activities that are attributed to its phenolic hydroxyl groups (hydrogen donors), which lowers the formation of hydroxyl peroxide [42,82].

A limited number of studies on the use of yucca and its products as an antioxidant in aquaculture have been done (Table 3). In this context, Wang et al. [42] recorded an improvement in the total antioxidants capacity (TAC) of mirror carp (*C. carpio*) with the dietary incorporation of YSE, particularly at 400 mg/kg diet. Furthermore, all fish groups that received YSE showed lower malondialdehyde (MDA) levels compared to the control group; meanwhile, no significant alteration in SOD was found with YSE supplementations. Moreover, the relative expressions of mRNA of CuZn-SOD, CAT, GPx1a, and Nrf2 were increased with YSE inclusion, in contrast to the mRNA relative expression of Keap1 compared to the control group. Regarding Nile tilapia, Abdel-Tawwab et al. [12] reported higher activities of SOD, CAT, and GPx, and a lower MDA value with YSE + yeast (*S. cerevisiae*) water additives at 1.0 g/L (*p* < 0.05). Similarly, Bae et al. [49] found that feeding olive flounder (*P. olivaceus*) on diets with Yucca meal at 1.5 mg/kg diet improved SOD and myeloperoxidase (MPO) compared to the control group. Furthermore, Amoah et al. [62] reported an enhancement in SOD of juvenile Amur catfish (*S. asotus*) that were fed diets with 0.4% SG, 0.05% YM, 0.05% BG, and 0.05% SG + BG compared with the fish fed the control diet. In addition, Angeles Jr. et al. [51] reported higher survival rates following a hypoxia challenge trial in Nile tilapia that were fed diets with *Y. schidigera* extract by 11% and with extracts mixture of 150 mg/kg *Q. saponaria* + 150 mg/kg *Y. schidigera* by 22% as compared to the control.

## 6. Yucca as a Natural Cleaner for Aquatic Water Quality

The accumulation of inorganic nitrogen compounds (NH_4_^+^, NH_3_, NO_2_^−^, HNO_2_, and NO_3_^−^) resulting from the feces of aquatic organisms, organic matter, and the leftover feed affects the reproduction, growth, and resistance of fish to stressful conditions [83,84]. Romano and Zeng [83] concluded that not only the ammonia emissions in the farms could harmfully affect the performance of aquatic animals but also in the ecosystem and outdoors, the accumulation of ammonia emissions deteriorates the survivability of decapod crustaceans. Specifically, exposure to NH_4_^+^ and NH_3_ (TAN) pollution can cause gill damage, anoxia, disruption of blood vessels and osmoregulatory activity (damage to the liver and kidneys), and a decrease in the effectiveness of the immune system [85]. Furthermore, the NH_4_^+^ ions contribute to an internal reduction of Na^+^, which in turn, increases the toxicity of NH_3_ [86]. These effects can reduce fish feeding activity, fecundity, performances, and survival, leading to a loss of production and massive economic losses [87]. Concurrently, the fluctuations between the water acidity (pH) and temperature affect the ratio of the formed unionized ammonia (NH_3_), which causes toxicity and harmful impacts on the ecosystem and aquatic organisms [84,88,89]. Severe ammonia toxicity induces several effects on the aquatic animals, including the decrease of feed consumption, deteriorated physiological functions, unstable breathing through the gills, oxidative stress, diminished immunity, and inflammatory features in the gills [90,91,92]. The metabolism of protein inside the body can also be deteriorated, which causes the consumption of high amounts of energy to balance the protein level in the fish body [52]. The main methods to lower the ammonia toxicity is to stop feeding, change the water, increase the level of dissolved oxygen, use nitrification-specific bacteria, or carefully include lime in the ponds [93,94]. Additionally, the application of yucca extract is also recognized as a powerful tool for reducing the ammonia level in aquaculture ponds [11,35]. Yucca is applied mainly to reduce the levels of ammonia emissions in aquaculture ponds due to its content of steroidal saponin fractions, which has surface-active properties and can bind to ammonia via glycol-component fractions [11,95]. The reduced levels of accumulated ammonia would result in the balance of protein metabolism in the fish body and a reduction in energy consumption [52]. Hence, the feed utilization, growth performance, and physiological status of aquatic species can be improved using yucca. Additionally, yucca application results in the enhancement of the antioxidative, immunological, and anti-inflammatory responses in several aquatic animals [11,42]. In this sense, yucca is an alternative approach to overcoming the excessive use of antibiotics for eco-friendly aquaculture. More specifically, the intensive conditions produce abundant amounts of ammonia emissions, which induces oxidative stress, immunosuppression, and inflammatory features in a fish’s body [42]. The inclusion of yucca extract improved the quality of rearing water and lowered the accumulated ammonia in the case of mirror carp [42], Nile tilapia (*O. niloticus*) [12,17], striped catfish (*P. hypophthalmus*) [52], and European seabass juveniles (*D. labrax*) [45]. Yucca extract has steroidal saponins and glycol with an active surface attributed to ammonia’s adsorption [34]. Correspondingly, the reduction in ammonia levels is attributed to the binding of ammonia with steroidal saponins and glycols or the transformation of ammonia to nitrite and nitrate [12,95]. Furthermore, the decreased level of NH_3_ is correlated with a decreased level of pH and water temperature [12]. In this context, Fayed et al. [16] elucidated that yucca extract reduced the level of pH in water. Concurrently, the level of NH_3_ was probably lowered due to the relative reduction in the level of pH in rearing water. Hence, it can be concluded that yucca extract’s approach is recommended to protect aquatic organisms from the toxicity of waterborne ammonia.

The overall results provide impressive outcomes regarding using *Y. schidigera* in terms of potential interest in open-flow pond aquaculture and closed aquaculture systems. Correspondingly, *Y. schidigera* can be complementary to biofloc technology, which can also improve the growth and immune status of farmed fish via improving water quality parameters [96].

**Table 1:** *Yucca schidigera*/saponin effects on the growth performance and feed utilization of aquatic species.

Reference	Aquatic Animal sp.	Supplementation Type	Supplementation Source	Trial Period–(BW0)	Applied Doses	Recommended Dose(s)	Impacts
Wang et al. [42]	Mirror carp (*Cyprinus carpio*)	Diet	YSE	8 weeks–(45.21 ± 0.43 g)	0, 200, 400 mg/kg	400 mg/kg	(+) FBW, WG
Peterman et al. [46]	Channel catfish (*Ictalurus punctatus*)	Diet	Commercial blend (One Current™)	3 months–(11 g)	0, 1.8, 3.6 g	3.6 g	(+) WG, SGR, (−) FCR
El-Keredy and Naena [47]	Nile tilapia (*Oreochromis niloticus*)	Diet	YSE	8 weeks–(20 g)	0.1, 0.14, 0.2%	0.1%	(+) WG, FI, FER, PER
Bae et al. [49]	Olive flounder (*Paralichthys olivaceus*)	Diet	YSM	8 weeks–(5.26 ± 0.17 g)	1.5 g/kg	1.5 g/kg	NS
Abdel-Tawwab et al. [12]	Nile tilapia (*Oreochromis niloticus*)	Water	YSE	8 weeks–(28–32 g)	0, 1 g/m^3^	1 g/m^3^	(+) FBW, WG, SGR, FI
Fayed et al. [16]	European seabass (*Dicentrarchus labrax*)	Water	YSE	45 days–(5.0 ± 0.5 g)	0.25, 0.50, 0.75 mg/L	0.75 mg/L	(+) FBW, WG, (−) FCR
Elkhayat et al. [45]	European seabass (*Dicentrarchus labrax*)	Diet	YSE	45 days–(5.0 ± 0.5 g)	0, 0.25, 0.50, 1.00 g/kg	1.00 g/kg	(+) FBW, WG, SGR, PER, (−) FCR
Njagi et al. [13]	Nile tilapia (*Oreochromis niloticus*)	Diet	YSM	10 weeks–(3.8 ± 0.05 g)	0, 0.1, 0.3, 0.5, 1.0, 2.0%	0.1, 0.14%	(+) WG, SGR, FER
Angeles Jr. et al. [51]	Nile tilapia (*Oreochromis niloticus*)	Diet	YSE-QS	6 weeks–(1.9 ± 0.08 g)	150 mg/kg	150 mg/kg *Quillaja saponaria* + 150 mg/kg YSE	(+) FBW, WG, SGR
Güroy et al. [52]	Striped catfish (*Pangasianodon hypophthalmus*)	Diet	YSE	12 weeks–(1.78 ± 0.05 g)	0, 0.075, 0.1, 0.15%	0.15%	(+) SGR, (−) FCR
Couto et al. [70]	European sea bass (*Dicentrarchus labrax*)	Diet	Soya-saponin	59 days–(27± 0.01 g)	0, 1, 2 g/kg	-	(−) Maltase and alkaline phosphatase activity, (+) intestinal inflammations
Couto et al. [69]	Gilthead sea bream (*Sparus aurata*)	Diet	Soya-saponin	48 days–(112± 5 g)	0, 0.1, 0.2%	-	NS
Chikwati et al. [67]	Atlantic salmon (*Salmo salar*)	Diet	Soya-saponin	80 days–(270 g)	2 g/kg	-	(−) FI, ADC
Chen et al. [48]	Japanese flounder (*Paralichthys olivaceus*)	Diet	Soya-saponin	56 days–(2.58 ± 0.01 g)	0, 0.8, 3.2, 6.4 g/kg	Lower level <0.8 g/kg	High level (6.4 g/kg) resulted in (−) WG, FI, FER
Gaber [33]	Nile tilapia (*Oreochromis niloticus*)	Diet	YSE	6 months–(14.2 ± 2.9 g)	0.075	0.075	(+) WG, SGR, ADC, whole-body protein content, (−) lower whole-body lipid content
El-Saidy and Gaber [53]	Nile tilapia (*Oreochromis niloticus*)	Diet	YSE	30 weeks–(16.82 g)	0, 250, 500, 750, 1000, 1250, 1500 mg/kg	750 or 1000 mg/kg	(+) BW, WG, SGR, FER, PER, ADC
Kelly and Kohler [43]	Channel Catfish (*Ictalurus punctatus*)	Diet	YSE	12 weeks–(post-yolk-sac and 19 g)	0, 0.5, or 0.1 g/kg diet	1 g/kg	(+) WG, whole-body protein content, (−) lower whole-body lipid content
Francis et al. [44]	Common carp (*Cyprinus carpio* L.)	Diet	YSE	8 weeks–(19 g)	0, 150, 300 mg/kg	150 mg/kg	(−) FCR, (+) metabolic growth rate
Francis et al. [54]	Nile tilapia (*Oreochromis niloticus*)	Diet	QS	14 weeks–(50 g)	0, 150, 300 mg/kg	300 mg/kg	(+) WG, average values for energy retention, apparent lipid conversion
Tidwell et al. [50]	Channel catfish (*Ictalurus punctatus*)	Diet	YSE	10 weeks–(1.9 g)	0, 0.011, 0.11, 1.10 g/kg	Lower levels <1.1 g/kg	(−) WG, (+) FCR
Hernández-Acosta et al. [55]	Pacific white shrimp (*Litopenaeus vannamei*)	Diet	YSE-QS	40 days–(2.6 g)	0, 0.25, 0.50, 1.00, 2.00 g/kg	1.00, 2.00 mg/kg	(+) WG, (−) FCR
Yang et al. [11]	Pacific white shrimp (*Litopenaeus vannamei*)	Diet	YSE	100 days–(82 ± 0.02 g)	0, 0.1, 0.2, 0.3%	0.2%	(+) WG

(+) symbol refers to an increase, (−) symbol refers to a decrease, BW0—initial body weight, FBW—final body weight, WG—weight gain, SGR—specific growth rate, FI—feed intake, FER—feed efficiency ratio, PER—protein efficiency ratio, ADC—apparent digestibility coefficient, YSE—*Yucca schidigera* extract, YSM—*Yucca schidigera* meal, QS—Quillaja saponin, NS—not significant, FCR-feed conversion ratio.

**Table 2:** *Yucca schidigera*/saponin impacts on the immune performance of aquatic organisms.

References	Aquatic Animal sp.	Supplementation Type	Supplementation Source	Trial Period–(BW0)	Applied Doses	Recommended Dose(s)	Impacts
Wang et al. [42]	Mirror carp (*Cyprinus carpio*)	Diet	YSE	8 weeks–(45.21 ± 0.43 g)	0, 200, 400 mg/kg	400 mg/kg	(+) LYZ, C3, C4, TGF-β2 mRNA levels, (−) mRNA levels of TNF-α, IL-1β, and IL-6
Peterman et al. [46]	Channel catfish (*Ictalurus punctatus*)	Diet	Commercial blend (One Current™)	3 months–(11 g)	0, 1.8, 3.6 g	3.6 g	(+) SR%
El-Keredy and Naena [47]	Nile tilapia (*Oreochromis niloticus*)	Diet	YSE	8 weeks–(20 g)	0.1, 0.14, 0.2%	0.1%	(+) SR%
Bae et al. [49]	Olive flounder (*Paralichthys olivaceus*)	Diet	YSM	8 weeks–(5.26 ± 0.17 g)	1.5 g/kg	1.5 g/kg	(+) NBT, CSR
Abdel-Tawwab et al. [12]	Nile tilapia (*Oreochromis niloticus*)	Water	YSE	8 weeks–(28–32 g)	0, 1 g/m^3^	1 g/m^3^	(+) serum total lipids, TP, albumin, globulin, (−) AST, uric acid, creatinine
Fayed et al. [16]	European seabass (*Dicentrarchus labrax*)	Water	YSE	45 days–(5.0 ± 0.5 g)	0.25, 0.50, 0.75 mg/L	0.75 mg/L	(+) LYZ, WBCs, RBCs, hematocrit, hemoglobin, lymphocytes, neutrophils
Reham et al. [63]	Nile tilapia (*Oreochromis niloticus*)	Water	YSE	93 days–(51 g)	1 mg/m^3^	1 mg/L	(+) LYZ, WBCs, hematocrit, hemoglobin, lymphocytes, neutrophils
Amoah et al. [62]	Amur catfish (*Silurus asotus*)	Diet	YSM	8 weeks–(4.95 ± 0.05 g)	0.05%	0.05%	(+) LYZ
Njagi et al. [13]	Nile tilapia (*Oreochromis niloticus*)	Diet	YSM	10 weeks–(3.8 ± 0.05 g)	0, 0.1, 0.3, 0.5, 1.0, 2.0%	0.1, 0.14%	(+) LYZ, NBT
Güroy et al. [52]	Striped catfish (*Pangasianodon hypophthalmus*)	Diet	YSE	12 weeks–(1.78 ± 0.05 g)	0, 0.075, 0.1, 0.15%	0.15%	(+) PCV%, haemoglobin
Yang et al. [11]	Pacific white shrimp (*Litopenaeus vannamei*)	Diet	YSE	100 days–(82 ± 0.02 g)	0, 0.1, 0.2, 0.3%	0.2%	(+) serum protein content, acid phosphatase, alkaline phosphatase, nitric oxide synthase, glutamic–pyruvic transaminase, phenol oxidase
Yeh et al. [64]	Giant freshwater prawns (*Macrobrachium rosenbergii*)	Water	Saponin	168 h–(17.9 ± 2.7 g)	0, 0.3, 0.6, 0.9, 1.2 mg/L	0.9 mg/L	(+) total hemocyte count, (−) phenoloxidase activity, respiratory protein level

(+) symbol refers to an increase, (−) symbol refers to a decrease, YSE—*Yucca schidigera* extract, YSM—*Yucca schidigera* meal, LYZ—lysozyme activity, SR%—survival rate percentage, NBT—nitroblue tetrazolium, CSR—cumulative survival rate, TP—blood total protein, AST—aspartate aminotransferase, WBCs—white blood cells count, RBCs—red blood cells count, PCV%— packed cell volume.

**Table 3:** *Yucca schidigera*’s impacts on the antioxidants of aquatic organisms.

References	Aquatic Animal sp.	Supplementation Type	Supplementation Source	Trial period–(BW0)	Applied Doses	Recommended Dose(s)	Impacts
Wang et al. [42]	Mirror carp (*Cyprinus carpio*)	Diet	YSE	8 weeks–(45.21 ± 0.43 g)	0, 200, 400 mg/kg	400 mg/kg	(+) TAC, (−) MDA, SOD
Bae et al. [49]	Olive flounder (*Paralichthys olivaceus*)	Diet	YSM	8 weeks–(5.26 ± 0.17 g)	1.5 g/kg	1.5 g/kg	(+) SOD, MPO
Abdel-Tawwab et al. [12]	Nile tilapia (*Oreochromis niloticus*)	Water	YSE	8 weeks–(28–32 g)	0, 1 g/m^3^	1 g/m^3^	(+) SOD, CAT, GPx, (−) MDA
Amoah et al. [62]	Amur catfish (*Silurus asotus*)	Diet	YSM	8 weeks–(4.95 ± 0.05g)	0.05%	0.05%	(+) SOD
Angeles Jr. et al. [51]	Nile tilapia (*Oreochromis niloticus*)	Diet	YSE + QS	6 weeks–(1.9 ± 0.08g)	150 mg/kg	150 mg/kg	(+) SR post a hypoxia challenge

(+) symbol refers to an increase, (−) symbol refers to a decrease, YSE—*Yucca schidigera* extract, YSM—*Yucca schidigera* meal, TAC—total antioxidants capacity, MDA—malondialdehyde, SOD—superoxide dismutase, MPO—myeloperoxidase, CAT—catalase, GPx—glutathione peroxidase, SR—survival rate. QS- Quillaja saponin.

## 7. Concluding Remarks

An updated and exclusive collection of results about yucca’s beneficial effects as a phytogenic additive for clean aquaculture activity are presented in this article. Yucca can clearly enhance the quality of rearing water by reducing ammonia emissions that result from aquatic organisms due to its potential as a medicinal herb. As a water remedy, yucca removes excess ammonia and reduces the water toxicity impacts on fish performances. The enhanced water quality clearly provides clean environmental conditions for aquatic organisms. Since aquatic organisms are known for their high sensitivity to environmental stressors, yucca’s application can be considered an active substance for the “blue clean aquaculture industry.” Additionally, yucca showed growth-promoting effects when included as a dietary additive, with possible feed utilization potential. The enhancement of feed digestion and nutrient absorption activates the local intestinal immunity, which leads to improved immunity and high resistance against infectious diseases. There are direct and indirect effects implicated for both axes involved in the aims of the present work: yucca can directly achieve improved water quality but immunity and growth may be indirectly affected. The review analyzed and reconsidered the published papers and presented their analyses and conclusions accordingly. The controlled aquaculture conditions were created to operate fish cultivation under strict veterinary and animal welfare regulations that were established after several decades of research and accumulated knowledge. Unfortunately, the ecosystem faces several environmental fluctuations (e.g., global warming and water pollution), which impact aquaculture requirements (e.g., water quality). Additionally, the controlled farming procedures are still not fully applied in several countries, especially the developing countries due to the lack of resources and technical experience; this motivates more investigative efforts toward sustaining aquaculture activity. The overall performances of aquatic organisms that were treated with yucca as a dietary additive or a water cleaner encourage performing further studies to prove its mode of action based on biochemical and biological techniques. 

## Data Availability

The data that support the findings of this study are available from the corresponding author, upon reasonable request.
